# Analysis of the Clinicopathological Characteristics and Risk Factors in Patients with Lung Cancer and Chronic Obstructive Pulmonary Disease

**DOI:** 10.1155/2018/8398156

**Published:** 2018-02-14

**Authors:** Jian-Long Miao, Jing-Jing Cai, Xiao-Feng Qin, Rui-Juan Liu

**Affiliations:** ^1^Department of Respiratory Medicine, Shandong Jining No. 1 People's Hospital, Jining, Shandong 272000, China; ^2^Department of Respiratory Medicine, Shandong Juxian People's Hospital, Juxian, Shandong 276511, China

## Abstract

**Objective:**

To investigate the clinicopathological characteristics and risk factors in patients with lung cancer and COPD.

**Materials and Methods:**

We retrospectively reviewed the clinical data of 282 patients with lung cancer, including 174 and 108 patients with and without COPD, respectively. Information on age, sex, smoking status, and histologic type was obtained from medical records.

**Results:**

Lung cancer patients with COPD and those with the chronic bronchitis (CB) phenotype had higher smoking indices compared to those without COPD (723.95 ± 631.48 and 920.95 ± 712.93 versus 418.40 ± 506.84; *P* = 0.010; *P* = 0.001, resp.), and current smokers accounted for significantly higher proportions of lung cancer patients with COPD and the CB phenotype versus without COPD (51.15% and 63.74% versus 35.19%; *P* = 0.009; *P* = 0.001, resp.). Adenocarcinoma was significantly more common in lung cancer patients without versus with COPD (48.15% versus 35.63%; *P* = 0.037), whereas small cell lung cancer was more common in patients with COPD (23.56% versus 13.89%). Among patients with COPD, male sex (odds ratio [OR], 19.946; *P* < 0.001), current smokers (OR: 6.588; *P* = 0.001), and age ≥ 75 years (OR: 2.670; *P* = 0.008) were identified as high-risk factors.

**Conclusion:**

The risk factors for COPD among lung cancer patients were age ≥ 75 years, current smokers with the CB phenotype, and male sex.

## 1. Introduction

Lung cancer is the most common cause of cancer-related death worldwide [1]. Lung cancer is divided into non-small cell lung cancer (NSCLC) and small cell lung cancer (SCLC) by pathological type. NSCLC accounts for 75% of lung cancers and comprises two predominant subtypes, adenocarcinoma and squamous cell carcinoma (SCC), which constitute 40% and 25% of NSCLC cases, respectively. Small cell lung cancer (SCLC) accounts for approximately 15–18% of cases [2] and is characterized by a rapid doubling time, high growth fraction, and early widespread metastasis [3]. Because of the lack of specific symptoms, most cases of lung cancer are diagnosed in the middle and late stages.

Chronic obstructive pulmonary disease (COPD) is a disease characterized by persistent airflow limitation. The progressive development of such limited airflow and the presence of toxic gas particles or chronic inflammatory reaction enhancement in the airway and lungs can result in acute exacerbation of symptoms and complications affecting the severity of the disease and the prognosis of the individual. The lungs are vulnerable to airborne environmental factors, and tobacco smoke in particular is implicated in lung inflammation [4, 5]. A relationship between COPD and lung cancer is evident from epidemiological and clinical studies [6, 7]. Smoking and other noxious particles, such as biofuel smoke, which cause inflammation of the lungs, are important causes of COPD, and smoking is the most common risk factor of COPD worldwide. In addition, air pollution, occupational exposure, and indoor biofuel pollution are also major risk factors for COPD in many countries. The developments of COPD and lung cancer are related to similar genetic and biological characteristics [8–12]. In fact, lung cancer and COPD may represent different manifestations of the same disease, with the same underlying genetic predisposition, telomere shortening, mitochondrial dysfunction, and premature aging [13]. A previous study reported that patients with a substantial smoking history accounted for 15%–20% and 50–80% of COPD and lung cancer patients, respectively [14]. Research has shown that 40–70% of lung cancer patients also have COPD, and the risk of COPD is sixfold higher in lung cancer patients who smoke, with smokers having host susceptibility to both COPD and lung cancer [9, 15–21].

The incidence of cancer increases with age [22], with the mean age of onset being 66 years [23]. COPD principally occurs in smokers above 40 years of age, and its incidence is 2.5 times higher in those aged >60 years [24]. The lifetime risks of developing lung cancer are 17.2% and 11.6% for males and females among smokers, as compared to only 1.3% for males and 1.4% for females among nonsmokers [25]. Hence, both COPD and lung cancer are related to age, smoking, and sex. While many studies have explained the pathogenesis and relationship of coexisting COPD and lung cancer and the risk factors thereof, few studies have analysed the detailed clinicopathological characteristics of lung cancer patients with COPD. Accordingly, this study retrospectively analysed the clinicopathological characteristics of lung cancer patients with and without COPD, with the aim of determining the relationships between clinical characteristics and subtypes in lung cancer patients with COPD.

## 2. Methods

### 2.1. Study Population

We retrospectively reviewed the clinical data of 282 patients with lung cancer, including 174 patients with COPD and 108 patients without COPD, diagnosed at the Department of Respiratory Medicine, Shandong Jining No. 1 People's Hospital, China, between January 2014 and December 2016. Information on age, sex, smoking status, and lung cancer diagnosis and histologic type was obtained from the patients' medical records. We defined never smokers as adults who had never smoked or who had smoked fewer than 100 cigarettes in their lifetime, former smokers as those who had smoked at least 100 cigarettes but currently do not smoke, and current smokers as those who had smoked at least 100 cigarettes in their lifetime and who currently smoke [26].

According to the diagnostic criteria for lung cancer and COPD, all patients were classified into 2 groups: lung cancer patients with COPD and lung cancer patients without COPD.

### 2.2. Diagnoses

All patients with lung cancer were diagnosed by pathology; the specimen was obtained by bronchoscopy, computed tomography-guided lung biopsy, operation, or evaluation of the hydrothorax. Histologically, lung cancer was categorized as SCLC or NSCLC. NSCLC subtypes were divided into adenocarcinoma, SCC, or others.

We identified all patients with a COPD diagnosis according to the American Thoracic Society criteria [27].

### 2.3. Statistical Analysis

All data were statistically analysed using SPSS (version 17.0; IBM, Armonk, NY). Continuous variables were compared by unpaired *t*-tests, while discrete variables were compared using the Chi-squared test. Logistic regression analysis was performed to obtain the odds ratios (ORs) and 95% confidence intervals (CIs) for the factors associated with lung cancer patients with COPD. Numerical data are expressed as the mean ± standard deviation. All *P* values are two-sided, with statistical significance defined as *P* < 0.05.

## 3. Results

### 3.1. Clinical Characteristics of the Study Patients

Among all included patients with lung cancer, males accounted for 77.66%. Current smokers accounted for 45.04% of the total patients. On the other hand, 31.91% of the patients were never smokers. Adenocarcinoma, SCC, and SCLC accounted for 40.43%, 32.27%, and 19.86% of the total patients, respectively. Patients aged ≥75 and ≤60 years accounted for 23.05% and 25.89%, respectively (Table 1).

### 3.2. Differences in Clinicopathological Characteristics between Patients with and without Chronic Obstructive Pulmonary Disease

In patients without COPD, the mean age was 62.69 ± 10.34 years (range: 37–85), and 66.67% (72/108) were male. Among patients with COPD, the mean age was 64.79 ± 8.49 years (range: 41–84), and 84.48% (147/174) were male. There was no statistical difference in age (*P* = 0.274), but males were significantly more common among the COPD group (*P* = 0.001).

Patients aged ≥75 years and ≤60 years accounted for 28.16% and 22.41% of patients with COPD and for 14.81% and 31.48% of patients without COPD, respectively. Patients aged ≥75 years accounted for a significantly higher proportion of patients with versus without COPD (*P* = 0.010).

Regarding the smoking status of lung cancer patients with and without COPD, the mean smoking indices were 723.95 ± 631.48 and 418.40 ± 506.84 packages, respectively (*P* = 0.010). The proportions of lung cancer patients with versus without COPD who were defined as current, former, and never smokers were 51.15% versus 35.19%, 25.86% versus 18.52%, and 23.00% versus 46.30%, respectively. The proportions of current and never smokers were significantly different (*P* = 0.009 and 0.001, resp.), whereas no difference was seen for former smokers.

Several COPD phenotypes have been proposed, but only a few have been validated [28]. One of these COPD phenotypes is the chronic bronchitis (CB) phenotype, which is generally defined as patients with COPD who experience chronic cough and sputum production for *⩾*3 months per year for two consecutive years [29]. Out of 174 patients with COPD, 52.30% (91/174) were defined as having CB. The mean smoking indices of the lung cancer patients with the CB phenotype versus without COPD were 920.95 ± 712.93 versus 418.40 ± 506.84 packages (*P* = 0.001), whereas that of patients with COPD of another phenotype was 535.91 ± 487.45 (*P* = 0.358 versus patients without COPD). The proportions of lung cancer patients defined as current, former, and never smokers with the CB phenotype versus without COPD were 63.74% (58/91) versus 35.19% (*P* = 0.001), 26.37% (24/91) versus 18.52% (*P* = 0.183), and 9.89% (9/91) versus 46.30% (*P* = 0.001), respectively, while the corresponding proportions for patients with COPD of another phenotype versus those without COPD were 37.35% (31/83) versus 35.19% (*P* = 0.758), 46.04% (21/83) versus 18.52% (*P* = 0.258), and 37.35% (31/83) versus 46.30% (*P* = 0.215), respectively.

Among lung cancer patients with COPD, adenocarcinoma, SCC, SCLC, and other subtypes accounted for 35.63%, 35.06%, 23.56%, and 5.75% of cases, respectively. The corresponding proportions among lung cancer patients without COPD were 48.15%, 27.78%, 13.89%, and 10.19%, respectively. Lung cancer patients without COPD had a significantly higher rate of adenocarcinoma (*P* = 0.037), whereas patients with COPD had a significantly higher rate of SCLC (*P* = 0.048) (Table 1).

### 3.3. Multivariate Regression Analysis for COPD Adjusted for Sex, Smoking Status, and Age among Lung Cancer Patients

In the multivariate analysis of lung cancer patients with COPD compared to without COPD, male sex (OR: 19.946; 95% CI: 6.531–60.914; *P* < 0.001), current smokers (OR: 6.588; 95% CI: 2.119–20.483; *P* = 0.001), and age ≥ 75 years (OR: 2.670; 95% CI: 1.289–5.529; *P* = 0.008) were found to be significant independent risk factors (Table 2).

## 4. Discussion

Our research found that lung cancer patients with COPD had a higher smoking index compared to those without COPD, as well as a higher proportion of current smokers. On the other hand, lung cancer patients without COPD included a larger proportion of never smokers. Accordingly, among lung cancer patients with COPD, current smoking was a significant risk factor compared with never smoking (OR: 6.588). An association of sex with COPD was also observed among our lung cancer patients; male patients accounted for a significantly higher proportion of patients with versus without COPD, and male sex was a significant risk factor for COPD among lung cancer patients (OR: 19.946). The age of the patients was also an independent risk factor, with age ≥ 75 years being significantly associated with a higher risk of COPD than age ≤ 60 years (OR: 2.670). Moreover, the proportion of adenocarcinoma was significantly higher in lung cancer patients without COPD, whereas SCLC was significantly more common among those with COPD.

In our study, NSCLC accounted for 76.44% of cases among the lung cancer patients with COPD. NSCLC comprises two predominant subtypes, namely, adenocarcinoma and SCC; these subtypes constituted 35.63% and 35.06% of cases, respectively. SCLC comprised 23.56% of all lung cancers among patients with COPD. The proportions of SCC and SCLC were both higher in the lung cancer patients with versus without COPD. This finding may be due to the associations of the two subtypes of lung cancer and COPD with smoking. The smoking effect on SCLC has been reported to be twofold: (1) an indirect effect mediated through COPD and (2) a direct effect mediated by pathways other than COPD [10]. Smoking-induced lung damage shares similar genetic and biological characteristics with lung cancer [8, 30]. While the effect of concomitant COPD has not been fully examined with regard to the SCLC risk, large-scale studies examining the precise associations between smoking, COPD, and SCLC will help shed light on its pathogenesis.

Ytterstad et al. [31] found that females had a lower risk of lung cancer mortality than males, indicating that lung cancer is associated with sex. In accordance with these findings, our study found that males accounted for the majority of lung cancer patients, especially among patients with COPD (84.48% versus 66.67% in patients without COPD). However, further studies to elucidate these associations are warranted.

Moreover, we found that patients aged ≥75 years accounted for a significantly higher proportion of lung cancer patients with COPD than without. Lim et al. [32] found that lower emphysema grades and reduced FEV1/FVC were independent predictors of central location of lung cancer in patients with COPD, and we speculate that this finding may be related to the fact that patients aged ≥75 years tend to have poorer lung function than younger patients. However, the results and the mechanism behind our findings should be further studied in larger cohorts.

In conclusion, this study found that the proportions of male sex, smoking, and age ≥ 75 years were significantly higher among lung cancer patients with COPD than without COPD. Further, SCLC was significantly more common among lung cancer patients with COPD, whereas adenocarcinoma was significantly more common among lung cancer patients without COPD. Future large-scale, multicentre studies are warranted to confirm and expand on our findings.

## Figures and Tables

**Table 1:** Clinical characteristics of lung cancer patients with and without chronic obstructive pulmonary disease (*N* = 282).

Characteristic	Lung cancer without COPD, *n* (%)	Lung cancer with COPD, *n* (%)	*t*(*χ*^2^)	*P*
Age (years), mean	62.69 ± 10.34	64.79 ± 8.49	1.101	0.274
≤60	34 (31.48%)	39 (22.41%)	2.856	0.091
60–75	58 (53.70%)	86 (49.43%)	0.488	0.485
≥75	16 (14.81%)	49 (28.16%)	6.692	0.010
Smoking index (packages), mean	418.40 ± 506.84	723.95 ± 631.48	2.631	0.010
Sex				
Male	72 (66.67%)	147 (84.48%)	12.192	0.001
Female	36 (33.33%)	27 (15.52%)	-	-
Smoking status				
Current	38 (35.19%)	89 (51.15%)	6.861	0.009
Former	20 (18.52%)	45 (25.86%)	2.026	0.155
Never	50 (46.30%)	40 (23.00%)	16.660	0.001
Subtype				
Adenocarcinoma	52 (48.15%)	62 (35.63%)	4.334	0.037
SCC	30 (27.78%)	61 (35.06%)	1.616	0.204
SCLC	15 (13.89%)	41 (23.56%)	2.797	0.048
Other	11 (10.19%)	10 (5.75%)	0.834	0.168

COPD: chronic obstructive pulmonary disease; SCC: squamous cell carcinoma; SCLC: small cell lung cancer.

**Table 2:** Multivariate regression analysis adjusted for sex, smoking status, and age.

Characteristic	OR	95% CI	*P*
Female sex	Ref.	-	
Male sex	19.946	6.531–60.914	<0.001
Age			
≤60 years	Ref.	-	
≥75 years	2.670	1.289–5.529	0.008
60–75 years	1.293	0.733–2.281	0.376
Never smoker	Ref.	-	
Current smoker	6.588	2.119–20.483	0.001

OR: odds ratio; CI: confidence interval.

## References

[1] Ferlay J., Soerjomataram I., Dikshit R. (2014). Cancer incidence and mortality worldwide: sources, methods and major patterns in GLOBOCAN 2012. *International Journal of Cancer*.

[2] Früh M., de Ruysscher D., Popat S., Crinò L., Peters S., Felip E. (2013). Small-cell lung cancer (SCLC): ESMO clinical practice guidelines for diagnosis, treatment and follow-up. *Annals of Oncology*.

[3] Kalemkerian G. P., Gadgeel S. M. (2013). Modern staging of small cell lung cancer. *Journal of the National Comprehensive Cancer Network*.

[4] Walser T., Cui X., Yanagawa J. (2008). Smoking and lung cancer: The role of inflammation. *Proceedings of the American Thoracic Society*.

[5] Takahashi H., Ogata H., Nishigaki R., Broide D. H., Karin M. (2010). Tobacco smoke promotes lung tumorigenesis by triggering IKK*β*- and JNK1-dependent inflammation. *Cancer Cell*.

[6] Adcock I. M., Caramori G., Barnes P. J. (2011). Chronic Obstructive pulmonary disease and lung cancer: New molecular insights. *Respiration*.

[7] Sekine Y., Hata A., Koh E., Hiroshima K. (2014). Lung carcinogenesis from chronic obstructive pulmonary disease: Characteristics of lung cancer from COPD and contribution of signal transducers and lung stem cells in the inflammatory microenvironment. *General Thoracic and Cardiovascular Surgery*.

[8] Houghton A. M. (2013). Mechanistic links between COPD and lung cancer. *Nature Reviews Cancer*.

[9] Young R. P., Hopkins R. J. (2011). How the genetics of lung cancer may overlap with COPD. *Respirology*.

[10] Huang R., Yongyue W., Rayjean J. (2015). Associated links among smoking, chronic obstructive pulmonary disease, and small cell lung cancer: a pooled analysis in the international lung cancer consortiumth. *EBioMedicine 2*.

[11] Skillrud D. M., Offord K. P., Miller D. W. (1986). Higher risk of lung cancer in chronic obstructive pulmonary disease. A prospective, matched, controlled study. *Annals of Internal Medicine*.

[12] Tockman M. S., Anthonisen N. R., Wright E. C., Donithan M. G. (1987). Airways obstruction and the risk for lung cancer. *Annals of Internal Medicine*.

[13] Durham A. L., Adcock I. M. (2015). The relationship between COPD and lung cancer. *Lung Cancer*.

[14] El-Zein R. A., Young R. P., Hopkins R. J., Etzel C. J. (2012). Genetic predisposition to chronic obstructive pulmonary disease and/or lung cancer: Important considerations when evaluating risk. *Cancer Prevention Research*.

[15] Takiguchi Y., Sekine I., Iwasawa S., Kurimoto R., Tatsumi K. (2014). Chronic obstructive pulmonary disease as a risk factor for lung cancer. *World Journal of Clinical Oncology*.

[16] Peek R. M., Mohla S., DuBois R. N. (2005). Inflammation in the genesis and perpetuation of cancer: Summary and recommendations from a National Cancer Institute-sponsored meeting. *Cancer Research*.

[17] Brenner D. R., McLaughlin J. R., Hung R. J. (2011). Previous lung diseases and lung cancer risk: A systematic review and meta-analysis. *PLoS ONE*.

[18] Turner M. C., Chen Y., Krewski D., Calle E. E., Thun M. J. (2007). Chronic obstructive pulmonary disease is associated with lung cancer mortality in a prospective study of never smokers. *American Journal of Respiratory and Critical Care Medicine*.

[19] Peto R., Darby S., Deo H., Silcocks P., Whitley E., Doll R. (2000). Smoking, smoking cessation, and lung cancer in the UK since 1950: Combination of national statistics with two case-control studies. *British Medical Journal*.

[20] Fletcher C., Peto R., Tinker C., Speizer F. E. (1976). *The Natural History of Chronic Bronchitis and Emphysema: An Eight-year Study of Early Chronic Obstructive Lung Disease in Working Men in London*.

[21] De Torres J. P., Bastarrika G., Wisnivesky J. P. (2007). Assessing the relationship between lung cancer risk and emphysema detected on low-dose CT of the chest. *CHEST*.

[22] Kamo K.-I., Katanoda K., Matsuda T., Marugame T., Ajiki W., Sobue T. (2008). Lifetime and age-conditional probabilities of developing or dying of cancer in Japan. *Japanese Journal of Clinical Oncology*.

[23] Rosenberger A., Illig T., Korb K. (2008). Do genetic factors protect for early onset lung cancer? A case control study before the age of 50 years. *BMC Cancer*.

[24] Lundbäck B., Lindberg A., Lindström M. (2003). Not 15 but 50% of smokers develop COPD?—report from the obstructive lung disease in Northern Sweden studies. *Respiratory Medicine*.

[25] Villeneuve P., Mao Y. (1994). Lifetime probability of developing lung cancer, bysmoking status Canada. *Can. J. Public Health*.

[26] Schoenborn C. A., Adams P. F., Peregoy J. A. (2013). Health behaviors of adults. United States, 2008–2010. *Vital Health Stat*.

[27] Celli B. R., MacNee W., Agusti A. (2004). Standards for the diagnosis and treatment of patients with COPD: a summary of the ATS/ERS position paper. *European Respiratory Journal*.

[28] Celli B. R., Decramer M., Wedzicha J. A. (2015). An official american thoracic society/european respiratory society statement: research questions in COPD. *The European Respiratory Journal*.

[29] Kim V., Criner G. J. (2013). Chronic bronchitis and chronic obstructive pulmonary disease. *American Journal of Respiratory and Critical Care Medicine*.

[30] Roca M., Roca I. C., Mihaescu T. (2012). Lung cancer a comorbidity in chronic obstructive pulmonary disease. *Revista Medico-Chirurgicala a Societatii De Medici Si Naturalisti Din Iasi*.

[31] Ytterstad E., Moe P. C., Hjalmarsen A. (2016). COPD in primary lung cancer patients: Prevalence and mortality. *International Journal of Chronic Obstructive Pulmonary Disease*.

[32] Lim J., Shin K. M., Lee K. S. (2015). Relationship between emphysema severity and the location of lung cancer in patients with chronic obstructive lung disease. *American Journal of Roentgenology*.

